# Report of Committee on Dental Science and Literature

**Published:** 1896-10

**Authors:** J. M. Teal

**Affiliations:** Kendallville, Ind., Chairman


### COMMUNICATIONS.

### Report of Committee on Dental Science and Literature.

J. M. TEAL, KENDALLVILLE, IND., CHAIRMAN.

Read before the Indiana State Dental Society, Indianapolis, June 30, 1896.

We must all recognize the fact that the science of dentistry, like all other sciences, is an exact thing, and it is, therefore, with a feeling of great thankfulness that we see the effort being made upon this line by those who are giving their time and thought to the work.

Your committee will not attempt to cover the whole field of dental science and literature, or elucidate theories, but try to emphasize a few salient points, leaving to each individual the task of gathering the ripened grain in his own way, for his own strengthening.

"Esthetic Correction of Facial Contours," C. S. Chase, M.D., D.D.S., Tri-State meeting. The principal point claiming notice is the creation of new territory and the movement into it of the teeth at fault. A careful reading of the paper should be made, and if clinically considered watch the results of the radical displacement of the tissues.

M. H. Cryer, M.D., D.D.S., American Dental Association, gives a fine study of the maxillary bones, calls attention to the variation found in the antrum of Highmore, the size and position, and the frequency of the floor being formed to accommodate the apex of roots of the teeth, and cites a case where this order has been changed to allow the crowns to show in the antrum. This is not new, but interesting. That section devoted to the lower jaw is more complete, possibly not having so many phases. As a whole, the excellent style in which the matter is treated and shown makes it, by far, the most valuable paper on the subject.

"Prosthetic Dentistry, Glenoid Fossa, Movements of the

Mandible," W. E. Walker, D.D.S., read before the Southern Dental Association. This is a more elaborate attempt to follow the movements of the human maxillary, in the grinding of food than any writer previously has made. Look the matter up and see if the author's deductions are correct.

"The Effects of Oxidation," G. V. Black, D.D.S., S.C.D. The conclusion seems to be that the alloys should contain 65 per-cent silver; should be fresh cut; practice suggested to place the material in position before oxidation occurs.

We would call your attention to another paper by Dr. Black, read before the New York Society, last January. This paper is so completely at variance with the theories formerly taught and now held by so large a percent of the profession in regard to the old supposed maxim, "soft teeth, poor teeth," that we will not mar the author's argument by giving any detached portion of it, but express the hope that every member of this association who has not read the article will read it, and those who have will read it again. The author enters the domain of filling material also, in this paper. He shows by instrumental tests what we all knew but never considered, that the teeth are harder and stronger than gold, either hammered or cast. That fillings, the best that had come under his test, showed too great a want of solidity; explains why old non-cohesive fillings were tooth-savers only where four good walls held, and that when force was made upon the surface their diameters enlarged, while their solidity did not become greater. Cohesive gold, he shows, simply becomes more compact until a density of fifteen volumes has been reached, when expansion begins to be noticeable. Through all this runs the thread of fact that gold is not yet the ideal filling material, for the reason that we have not yet learned the secret of attaching it to the walls of the cavity in such a manner that each particle when it comes in contact with the tooth substance will "glue" itself, for the want of a better term, thereto, thereby taking its share of the strain when force is brought to bear upon the completed filling. Whether this can be done by electrosis remains to be seen.

Of the new local anæsthesia cataphoric method your committee will refrain from discussion, as we are to have a clinic demonstration during this meeting of this subtle agent.

Rosenthal suggests silver bands slipped over the teeth for the cure of pyorrhœa alveolaris.

E. S. Talbot, M.D., D.D.S., again gives us a valuable paper on " Dental and Facial Evidence of Constitutional Defects."

At the proper time during this meeting the committee would be pleased to have some expression from the association in regard to dental literature for the public press.

